# Coding of low-level position and orientation information in human naturalistic vision

**DOI:** 10.1371/journal.pone.0212141

**Published:** 2019-02-11

**Authors:** Jeppe H. Christensen, Peter J. Bex, József Fiser

**Affiliations:** 1 Department of Psychology, University of Copenhagen, Copenhagen, Denmark; 2 Department of Psychology, Northeastern University, Boston, Massachusetts, United States of America; 3 Department of Cognitive Science, Central European University, Budapest, Hungary; 4 Department of Brain and Cognitive Sciences, University of Rochester, Rochester, New York, United States of America; Johns Hopkins University, UNITED STATES

## Abstract

Orientation and position of small image segments are considered to be two fundamental low-level attributes in early visual processing, yet their encoding in complex natural stimuli is underexplored. By measuring the just-noticeable differences in noise perturbation, we investigated how orientation and position information of a large number of local elements (Gabors) were encoded separately or jointly. Importantly, the Gabors composed various classes of naturalistic stimuli that were equated by all low-level attributes and differed only in their higher-order configural complexity and familiarity. Although unable to consciously tell apart the type of perturbation, observers detected orientation and position noise significantly differently. Furthermore, when the Gabors were perturbed by both types of noise simultaneously, performance adhered to a reliability-based optimal probabilistic combination of individual attribute noises. Our results suggest that orientation and position are independently coded and probabilistically combined for naturalistic stimuli at the earliest stage of visual processing.

## Introduction

It has been long known that luminance information arrives from the retina to the visual cortex in a topographically organized arrangement [1,2], yet the nature, precision and the underlying mechanism by which this information is coded and used during natural object and scene perception is unclear. One typical approach to this problem focuses on the perceptual tolerance of the human visual system to either local element position (i.e. topographical jitter) or local element orientation noise in a range of laboratory-generated simple artificial stimuli. The computational rationale behind such investigations is that identifying these limits would be instructive for understanding natural vision, since those fixed limits of precision would define what information about the attributes is accessible and used during higher perception of more complex visual forms and shapes [3–5].

Previous investigations have found that for isolated lines or Gabor-patches, human sensitivity to orientation differences is around 1° [6,7]. For positions, the situation is less univocal. Using isolated Gabor and Gaussian patches, Levi and co-workers [8,9] found that the visual system is very robust to positional jitter of individual elements. In normal vision, this robustness persists over a wide range of contrasts, carrier orientations and polarities, both in the fovea and in the periphery [10]. Such robustness might imply low sensitivity to position changes. However, humans exhibit high sensitivity to positional deformations of radial and curved patterns [11,12], and to line segments in Vernier acuity type tasks [13], indicating that coding precision depends on stimulus configuration. Furthermore, position sensitivity is affected by the presence and proximity of noise elements. Observers are highly sensitive to the spacing between contour elements and noise elements when asked to detect a contour embedded in noise [14], and this effect of separation is also influenced by the orientations and orientation jitter of neighbouring elements [15,16], as well as by spatial attention [17]. Thus, depending on the stimulus and task configuration, position information might be either precisely or more loosely encoded, and this precision can even change with increasing experience during the task [18].

The stimulus- and task-dependence of positional sensitivity highlights the fact that assessing sensitivity in studies employing highly artificial stimuli of a few Gabor-elements in isolation might not generalize well to object perception under natural viewing conditions. Indeed, psychophysical [19,20] and physiological [21,22] studies reported that for more complex visual stimuli, human behavioural, as well as neural responses, are distinctively different from those measured with simple stimuli. This calls for experimental paradigms that can assess and compare sensitivity to changes in basic visual attributes rigorously in complex inputs under various conditions.

In a recent study, we provided such a paradigm and investigated the precision of human orientation coding using naturalistic stimuli. These novel stimuli consisted of 700 Gabor-elements spatially arranged according to the higher-order configuration of images from three classes, simple geometric arrangements, natural objects, and fractals. We found that the just-noticeable difference (JND) for local element orientation noise followed a dipper function across increasing levels of added pedestal noise [23], indicating the presence of a sensory threshold and confirming previous findings that used simple isolated Gabor-elements arranged in a lattice [24]. Moreover, the absolute levels of sensitivity were systematically determined by the complexity and object familiarity of the presented image class. Thus, prior expectancy dynamically affected orientation coding in the visual processing stream.

In the present study, we employed the experimental procedure of Christensen, Bex & Fiser [23] to address three outstanding questions. First, are the orientation and position of low-level visual elements encoded similarly or in a fundamentally different manner in humans? Second, how is information concerning different attributes combined? Third, is there an expectancy-based effect on position coding in humans like what was found with orientation coding? In Experiments 1 and 2, we found that variances of local elements’ orientation and position in naturalistic stimuli were distinctly encoded by different mechanisms. At the same time, higher-order properties of the stimulus scene affected the absolute level of coding precision similarly for orientation and position. In Experiments 3 and 4, we found that variances of different attributes were independently encoded and optimally integrated. We conclude that the visual system probabilistically combines attributes of the visual input together with previously stored internal information for coding even very low-level aspects of the input image.

## Methods

### Participants

All naïve participants were recruited from the Central European University (b/w 18–25 years of age, sex unknown) and were paid 6€ per hour of testing. Sample-sizes for Experiments 1 to 3 were chosen based on earlier studies [23], and all experiments in this study were approved by the Hungarian Central Ethical Review Committee for Research in Psychology and performed in accordance with the relevant guidelines and regulations. Informed written consents were obtained from all participants prior to testing.

### Stimuli

Stimuli were generated by a method similar to that used in Christensen, Bex, & Fiser [23] based on and extending the method originally introduced by Kingdom, Hayes, and Field [25], in which large numbers of randomly-positioned Gabor-elements were used to create noise patterns. Our method first decomposes an arbitrarily chosen image into its local representation by convolving it with a bank of wavelet (Gabor) filters, in a process similar to what is thought to occur in the mammalian visual cortex [26]. Next, a selected subset of the resulting Gabor-elements is used to reconstruct a degraded version of the original image. Since the resulting image contains only Gabor-elements with known position, contrast, phase, spatial frequency, and orientation information, human sensitivity to perturbations of these attributes can be systematically assessed.

**Fig 1:**
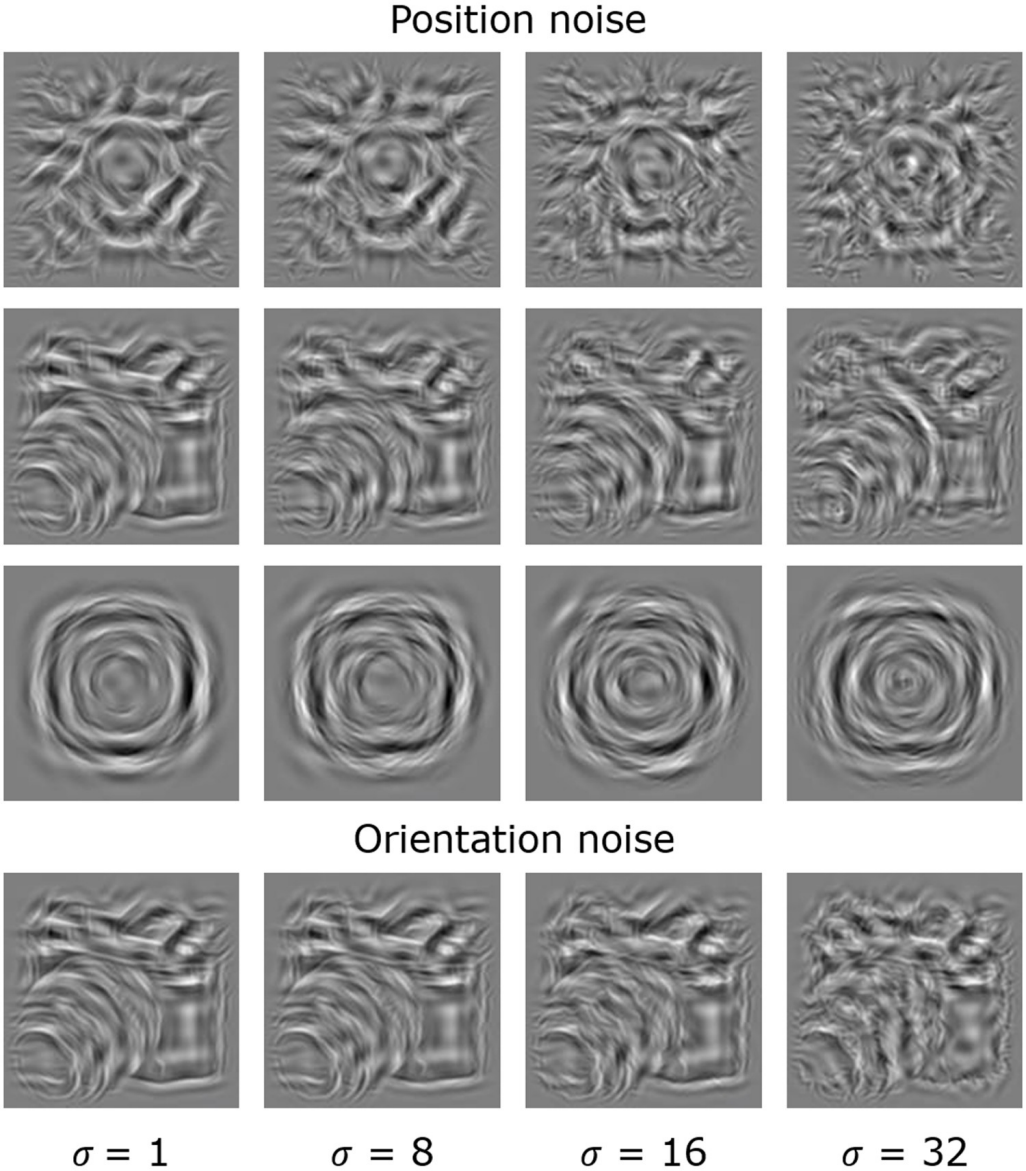
Sample stimuli. Fractal (1st row), object (2nd row), and circular (3rd row) image classes at four levels of Gaussian noise (σ) added to the position of each element. The same object with added orientation noise using the same four σ values measured in degrees produces similar appearance to those with position noise (4th row).

We used stimuli belonging to three classes: 1) naturalistic objects; 2) fractal patterns and 3) simple circular patterns (see Fig 1).

40 object and fractal source images were selected from a database (royalty-free object images from Hemera Photo-ObjectsTM Volume III, 2003, Hemera Technologies Inc.), scaled to 512 x 512 pixels, and then analyzed with a bank of steerable wavelet filters at three spatial frequency scales and four orientations. The filters were defined as:

$$F_{\theta }=\frac{1}{\sqrt{2\pi }\sigma_{x}\sigma_{y}}\text{exp}\left[-\frac{x^{2}}{2\sigma_{x}^{2}}-\frac{y^{2}}{2\sigma_{y}^{2}}+i2\pi \omega_{s}s_{\theta }\right]$$ (1)

The center of the spatial frequency bands (*ω*_*s*_) were 8, 16 and 32 c/image, (wavelength: *λ*_*s*_ = 64, 32 or 16 pixels, respectively), orientations (θ) were spaced at 45° intervals from 0° to 135°, *s*_*θ*_ = *x*cos_*θ*_ + *y*sin_*θ*_, and the standard deviation of the Gaussian envelope were *σ*_*x*_ = *σ*_*y*_ = 0.25*λ*_*s*_. The images were analyzed separately at each spatial frequency and steering orientation. The response magnitude of each filter, R, at each point in the image is:

$$R_{\theta_{\left(x,y\right)}}=\sqrt{{real}_{\left(x,y\right)}^{2}+{imag}_{\left(x,y\right)}^{2}}$$ (2)

The phase, ϕ in the image is:

$$\phi_{\theta_{\left(x,y\right)}}=a\text{tan}2({real}_{\left(x,y\right)},{imag}_{\left(x,y\right)})$$ (3)

The interpolated orientation, $\hat{\theta }$, at each point in the image is calculated as:

$${\hat{\theta }}_{\left(x,y\right)}=a\text{tan}2(\sum_{\theta =0}^{135}R_{\theta_{\left(x,y\right)}}\text{sin}(\theta ),\sum_{\theta =0}^{135}R_{\theta_{\left(x,y\right)}}\text{cos}(\theta ))$$ (4)

Prior to experiments, this analysis was performed on each image, yielding a pixel-by-pixel record of the local amplitude, phase and orientation information of the image at each spatial frequency. Next, this pixel-by-pixel record was used for each image individually to select a pre-specified number of Gabor-elements: 100, 200 and 400 elements for the 8, 16 and 32 c/image bands, respectively. In each band, selection proceeded by choosing the next available Gabor from the pixel-by-pixel record with the highest amplitude of Michelson contrast with one extra condition: no two Gabor-elements within one scale could be selected with a center-to-center spacing smaller than 2*σ*. This condition assured that there was no visible overlap between the selected Gabors in the image and that the overall spatial density of Gabor-elements in the three stimulus classes were matched (see next section). Finally, all selected Gabor-elements across all scales were superpositioned to obtain a reconstructed synthetic naturalistic version (the stimuli) of the original image. Stimuli of simple circular patterns consisted of concentric rings (see Fig 1).

For these, the orientation of each Gabor-element was determined by its position relative to the centre of the image, *θ*_(*x*,*y*)_ = atan2(−*y*,*x*). The position of each element was drawn from a multimodal distribution with the modes of the distribution at the concentric rings at increasing distances from the centre. Prior to presentation, the intensity profile of each stimulus in the image classes was adjusted to ensure that all stimuli had the same RMS contrast. Furthermore, all stimuli had frequency spectrums close to that found in natural images (see Figure B in S1 File) [27–29].

While stimuli were equated on low-level statistics by the stimulus generation method, the classes consisted of stimuli with different high-level complexities. Here, complexity is defined as the reciprocal of low-level predictability, i.e. how well the attributes of one local element (Gabor-element) can be inferred when knowing the basic attributes of neighbouring ones. Circular patterns represent the least complex image class since the attributes of a given Gabor-element is relatively well constrained by knowledge of neighbouring ones without the need for higher-level knowledge about the scene. In contrast, objects and fractals contain long disjointed edges that are sampled sparsely by our stimulus generation method. Thus, the exact shape and superposition of these features are locally less predictable than the features of the ordered Gabor-elements in the circular patterns. In addition, since stimuli from the object class consist of familiar natural objects, the predictability of local orientation and position information of these images should be facilitated by higher-level prior expectancy.

Stimuli were presented on a 27” iMac with a mean luminance of 50 cd/m^2^, a refresh rate of 60Hz, and a resolution of 1920-by-1080 pixel. The display measured 66° horizontally (65.0cm), 35° vertically (31.3cm), with a distance of 50 cm from the observer, in a semi-lit room. Thus, each stimulus image extended 19.6° of visual angle horizontally and 16.9° of visual angle vertically, with each horizontal pixel extending 0.038° and each vertical pixel extending 0.033°. The RGB monitor settings were adjusted so that the luminance of green was twice that of red, which in turn was twice that of blue. This shifted the white-point of the monitor to 0.31, 0.28 (x,y) at 50 cd/m^2^. A bit-stealing algorithm [30] was used to obtain 10.8 bits (1785 levels) of luminance resolution on the RGB monitor. All stimuli were presented with raised cosine spatio-temporal envelopes with edges smoothed over 0.5°. Image contrast was ramped on and off over 3 video frames (50 ms).

To ensure that stimuli were in fact equated on low-level statistics, we evaluated and compared the orientation information distribution, the spatial frequency characteristics, and the spatial density profiles for each stimulus from each image class. We found no differences between the image classes (see Figures A and B in S1 File).

### Task procedure

**Fig 2:**
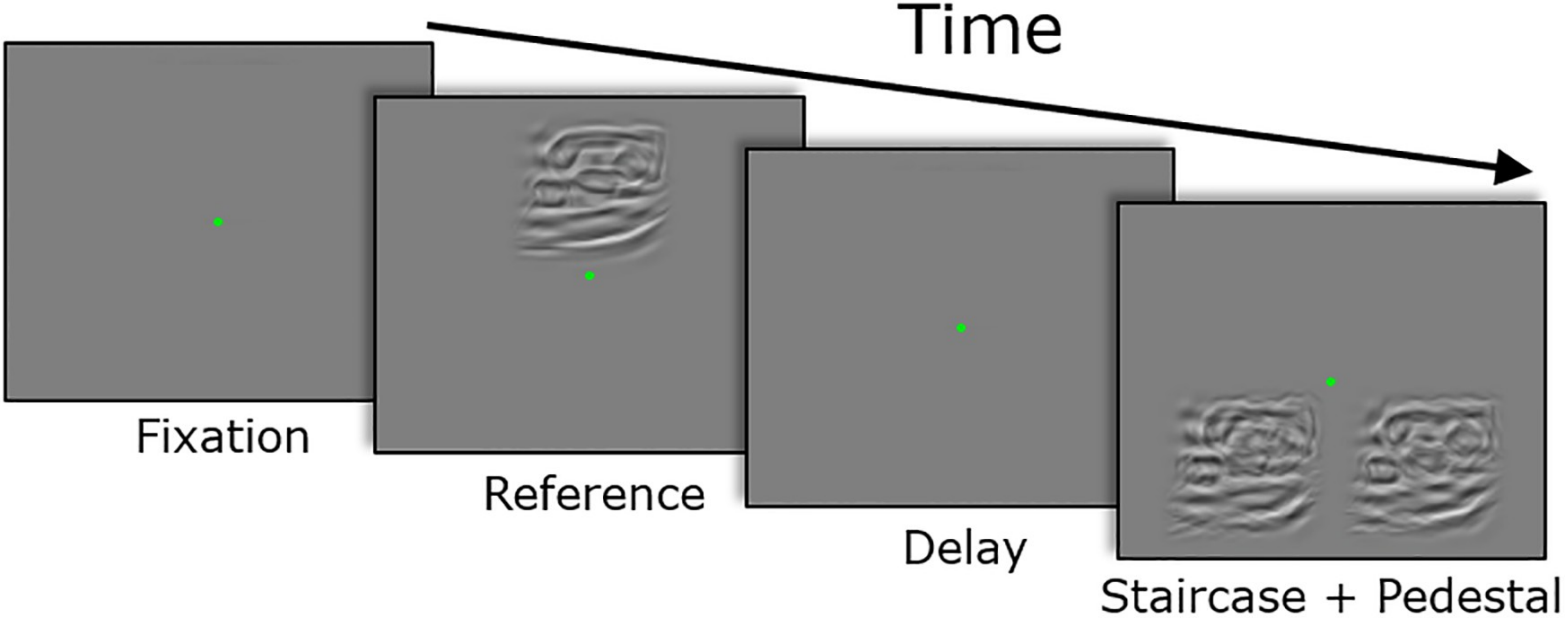
Trial procedure for estimating the JNDs. After fixation, the participants first viewed a noiseless Reference image followed by the Pedestal and Staircase image, randomly positioned to the left or right of the fixation dot. The task was to report back which of the stimuli contained the larger amount of noise (i.e. when Δ*σ* became perceivable). The example in the Fig shows an object class stimulus (an iron) and the Staircase image is on the left.

For the first three experiments, we used an equivalent noise paradigm to measure the just-noticeable difference (JND) for position or orientation noise (Experiments 1–2), or for a combination of position and orientation noise (Experiment 3). Participants first viewed a noiseless Reference image for 800 ms above a fixation point (Fig 2).

After a 500-ms delay with a grey background screen, a Pedestal and a Staircase image appeared together for 800 ms below the fixation point, randomly alternating sides across trials. The Pedestal image contained all the Gabor-elements of the Reference image, but each Gabor-element was randomly perturbed by position noise orthogonal to its orientation (Experiments 1–3); by orientation noise (Experiments 2–3); or by a combination of position and orientation noise (Experiment 3). The pedestal noise was drawn from a zero-mean Gaussian distribution with a standard deviation of *σ*_p_ (see next section for specific pedestal levels). The Staircase image was identical to the Pedestal image, except that the standard deviation of the normal distribution from which the perturbation of each element was drawn was increased by Δ*σ* under the control of a 3-up-1-down staircase, which constrained each observer’s ideal accuracy to 75%. In each trial, after freely saccading between the images, the observer’s task was to make a 2AFC decision whether the noisier (Staircase) image was on the left or on the right.

Visual feedback was provided with a photometrically isoluminant fixation mark, which was green following a correct response or red following an incorrect response. The observed JNDs were estimated as the 75% correct point of cumulative normal psychometric functions using one lapse-rate per stimulus. The JND for each experimental condition (pedestal level, image class, and noise type) were determined based on 50 staircase trials.

**Fig 3:**
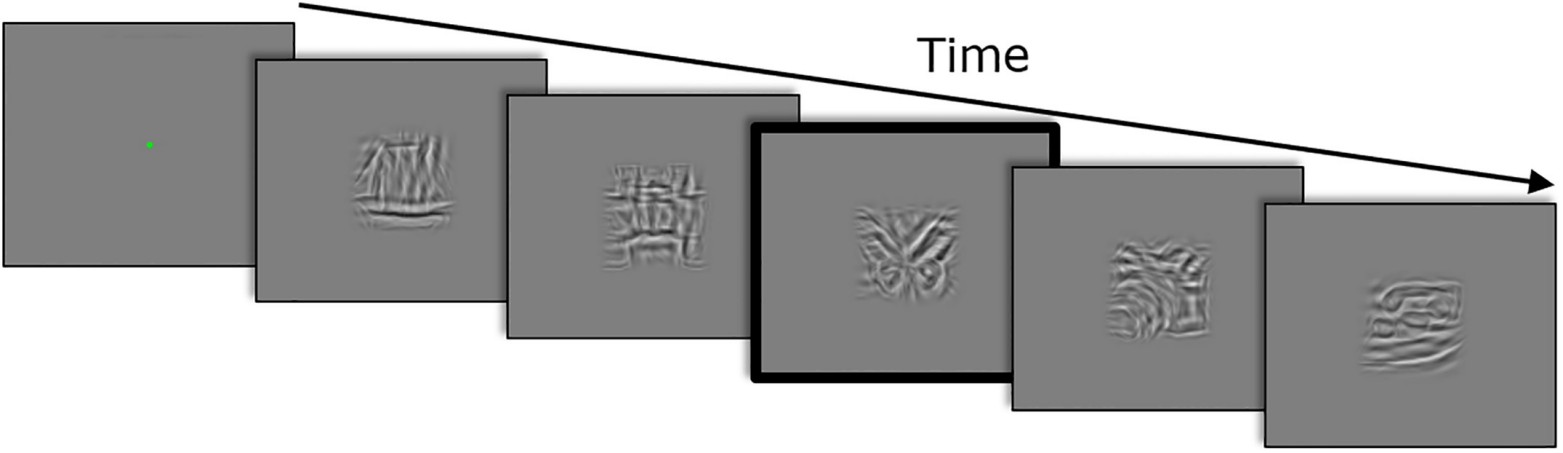
One experimental trial of Experiment 3 estimating noise discriminability performance. Participants viewed five consecutively presented object stimuli with an odd-one-out stimulus (red outline) perturbed with position noise while the four other stimuli are perturbed with orientation noise. Each stimulus was presented for 800 ms, and the participants had to respond whether they detected an odd-one-out stimulus in the stream.

In Experiment 4, we adapted the method of Cohen, Nakayama, Konkle, Stantić, & Alvarez [31], and tested how well participants could distinguish whether object class stimuli were perturbed by orientation or position noise. Participants were asked to detect if one of five consecutively presented stimuli was an odd-one-out—that is, a stimulus perturbed by adding position noise embedded in a series of stimuli with orientation noise or vice versa. If the perceptual effect of one noise type is stronger (i.e. consciously more visible) than the other, participants should correctly detect the odd-one-out stimulus. Conversely, if participants cannot tell apart the two types of perturbation, any difference in behavioural measures with the two noise manipulations must be due to low-level implicit mechanisms. On each trial, the participants saw five, sequentially presented and centrally positioned, stimuli from the object image class (see Fig 3).

Each stimulus had the same dimensions as stimuli in Experiment 1 to 3 and was randomly chosen among the 40 exemplars without replacement. For half of trials, the stimuli were all perturbed with the same type of noise (i.e. no odd-one-out present), for the other half, four of the stimuli had one noise type manipulation and the fifth was perturbed by the other noise type representing the odd-one-out stimulus. The noise levels were the same for the five stimuli within each trial, but varied randomly across trials between *σ* = 4, *σ* = 8, *σ* = 16. When an odd-one-out stimulus was present, it was either in the second, third, or fourth position in the stream, appearing in each position equally often across trials. Each stimulus in a trial was displayed for 800 ms (as in the main experiment) with a 100-ms delay between presentations, and each trial was initialized with a centrally positioned fixation dot presented for 500 ms. At the end of each trial, participants had to indicate by pressing either “1” (yes) or “2” (no) whether the noise appeared different in one of the presented stimuli. Participants were told that the noise in the presented images might differ, but details of the noise types were not explicitly revealed prior to testing. The 6 experimental conditions were odd-one-out noise present/not present combined with three noise-levels.

Note that in this setup, the four stimuli in a trial with the same type of noise and the explicit task provide a strong context that helps discriminating the fifth odd stimulus. The magnitude of difference sufficient to perform well in such a setup is much smaller than what would be required to produce an effect in Experiment 2 and 3 (see above), where this difference in the stimulus appearance should automatically initiate a very different processing of stimuli during the judgment of the level of noise.

### Experimental design and statistical analysis

#### Measuring noise discrimination sensitivity

In Experiment 1, participants position noise discrimination sensitivity were investigated for fractals, natural objects, and simple circular patterns. Trials with different image classes (3 levels) were blocked and counterbalanced across participants, and in each block, the pedestal levels were randomly interleaved in log steps from 1 to 32 (6 levels) trial-by-trial.

In Experiment 2, within-participant differences in position and orientation coding were assessed by presenting stimuli from the object class perturbed by either orientation or position noise in separate blocks (2 levels), across eight levels of pedestal noise ranging from *σ*_p_ = 2 to *σ*_p_ = 32 (in units of degrees or pixels, respectively). Note that in our experimental setup, the angular displacement of the Gabor-elements by one degree corresponded to 0.38mm of arc length, while each pixel in our display was approximately 0.34mm wide. This ensured that the physical metrics of orientation noise (in degrees) and position jitter (in pixels) were roughly equal.

**Fig 4:**
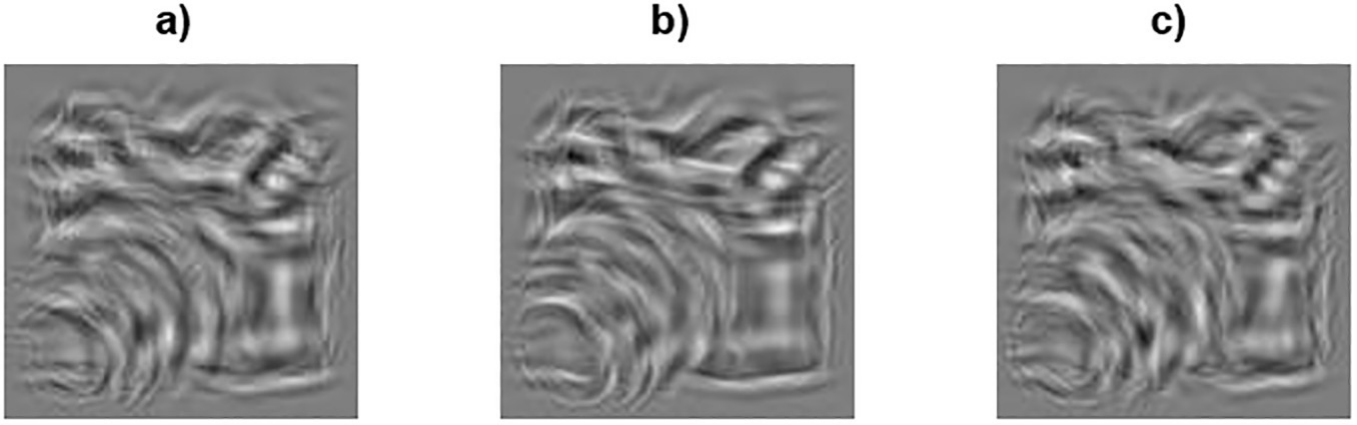
Examples of perturbing an object class stimulus with different noise types. (A) position noise, (B) orientation noise, and (C) combined noise. The noise level of each attribute (position and orientation) is *σ*^2^ = 64.

In Experiment 3, participants discriminated between stimuli either perturbed by position noise or orientation noise alone (single-noise conditions), or by an equal combination of the two (Fig 4).

In the combined noise condition Gabor-elements were perturbed on their orientation and position simultaneously by values drawn from two independent zero-mean Gaussian distributions, defined in pixels and degrees. The variance of the pedestal and staircase noise was the same for each attribute (i.e. $\sigma_{\text{p}}^{2}=\sigma_{\text{p},\text{position}}^{2}=\sigma_{\text{p},\text{orientation}}^{2}=\left\{16,64,256\right\}$ and Δ*σ* = Δ*σ*_position_ = Δ*σ*_orientation_). Trials with individual and combined noise were blocked and counter-balanced across participants, and only stimuli from the object class were presented. Thus, the two independent variables were noise type (3 levels) and pedestal noise (3 levels).

Values of JNDs are presented as mean ± the 95% confidence interval (CI). The level of significance between experimental levels and JNDs were tested with repeated-measures 2-way ANOVAs and significance between modelling parameters (see next section) and image classes were tested with repeated-measures 1-way ANOVAs. Significance between parameter estimates of different models were tested with two-tailed paired t-tests.

#### Measuring perceptual effect of noise

Detection rates in Experiment 4 are based on the number of correctly identified odd-one-out trials (true positives, TP) together with the number of correctly rejected non-target trials (true negatives, TN) with respect to the total amount of trials, N (detection rate = (TP + TN)/N). Given the 2AFC design, responses are binomially distributed with a probability of success, *P*, equal to the detection rate. In the case of pure guessing, *P* would not be significantly different from 50%. We tested each response distribution for each experimental condition (noise level σ: 4, 8, 16; noise type: orientation, position) with the two-sided exact binominal test [32] against a hypothesised probability of success of P = 50%.

### Maximum likelihood prediction of combined noise coding

The JNDs for combined noise in Experiment 3 are predicted by a maximum likelihood estimate (MLE), which assumes that noisy perturbations of each attribute represents independent perceptual cues for the variance discrimination task [33]. The MLE is computed using a simple probabilistic rule: in the combined case, the precision that each attribute is encoded by individually is equal to the uncertainty of the same cue relative to the other cue. Thus, the MLE of the JND for combined noise (${\Delta \sigma }_{MLE}^{2}$) is calculated as the average of the individual cues (i.e.$\;{\Delta \sigma }_{pos}^{2}$ and ${\Delta \sigma }_{ori}^{2}$) weighted by each cue’s reliability, where reliability is defined by the reciprocal of the uncertainty (i.e. coding precision):

$${\Delta \sigma }_{MLE}^{2}={\left(\frac{1}{{\Delta \sigma }_{pos}^{2}}+\frac{1}{{\Delta \sigma }_{ori}^{2}}\right)}^{-1}$$ (5)

### Computational model

Results of JNDs in pedestal-based experiments can be presented both in units of standard deviation (SD) and in variance. However, these units transform the data differently, which can obscure a clear assessment of the effect of treatment and the presence of a sensory threshold [34]. For example, if the JND is Δ*σ* = 5 for a pedestal of *σ*_p_ = 8 and Δ*σ* = 4 for a pedestal of *σ*_p_ = 16, this would indicate a “dip” in the JND-versus-pedestal curve—that is, a curve in which the JND at low pedestal noise values would first fall as the pedestal noise increases, and it would rise proportionally with the increase in pedestal noise only at higher pedestal noise levels. In contrast, expressed in variance units, the JNDs are Δ*σ*^2^ = (5 + 8)2 − 8^2^ = 105 and Δ*σ*^2^ = (4 + 16)2 − 16^2^ = 144, respectively, and since variance scales quadratically, the dip in units of SD get masked by the relative larger values of pedestal SDs. To this end, when it was relevant, we plotted our results in both units of SD and variance, but modelled and discussed effects in terms of differences in the parameters of the noisy, inefficient (but otherwise ideal) observer model (NIO) of Solomon, Morgan, and colleagues [24,35,36] in order to assess significant differences.

The NIO model makes explicit predictions derived from signal detection theory. Briefly, the model assumes that the observer sample *M* independent elements from each of the two test stimulus images and compute sample variances by comparing the attribute of the elements with those from a noisy template. The noisy template implies that during perception, sample elements of the stimulus are perturbed by zero-mean Gaussian internal noise with variance $\sigma_{int}^{2}$. Response accuracy of the selection can then be predicted by comparing the two samples variance with an *F*-test, where the variance of the external noise in the Pedestal image is $\sigma_{p}^{2}$, while in the Staircase image, it is (*σ*_p_ + Δ*σ*)^2^. The value of Δ*σ* is changing adaptively on each trial to reach an average response accuracy of 75%. Thus, the probability of correct choice on each trial in our experiment is predicted by [35]:

$$P=1-(1-2\delta )F[\frac{\sigma_{p}^{2}+\sigma_{int}^{2}}{{\left(\sigma_{p}+\Delta \sigma \right)}^{2}+\sigma_{int}^{2}}]+\delta ,$$ (6)

where *F* is the cumulative *F*-distribution with *M* − 1 and *M* − 1 degrees of freedom, and the lapse rate, *δ*, is estimated for each participant and stimulus class during fitting of the psychometric functions.

In general, a threshold implies that any variability of an attribute below the threshold value is discounted and therefore, indistinguishable. The internal noise of Eq 1 already represents a threshold: If $\sigma_{p}^{2}\;<<\;\sigma_{int}^{2}$, the internal noise will dominate the percept. However, $\sigma_{int}^{2}$ is an additive noise and previous findings have confirmed that for some attributes (e. g. orientation), achieving the best match between human and model performance requires the addition of a “hard” *sensory threshold* to the model [23,24])—that is, a gating mechanism that allows external variability to factor in only when it exceeds the threshold level.

In our 2AFC task, such sensory threshold can be formally implemented by an ideal observer model with three conditions, each predicting the probability of correct choices (*P*_1_, *P*_2_, and *P*_3_) under particular circumstances. The first condition assumes that the sample variances of both the Pedestal and Staircase image are below the sensory threshold (i.e. the variances are zeroed), in which case the probability of correct would be 50% (pure guessing). Formally, this can be modelled by assuming that the percept of both the Pedestal and Staircase image consist of intrinsic noise with a variance equal to the threshold variance, $\sigma_{thres}^{2}$:

$$P_{1}=1-(1-2\delta )F[\frac{\sigma_{thres}^{2}}{\sigma_{thres}^{2}}]+\delta ,$$ (7)

The second condition assumes that the sample variance from the Pedestal image is below the threshold, but the sample variance from the Staircase image has exceeded the threshold due to the added staircase noise. In this case, the response accuracy is determined by comparing the sample variance of the Staircase image with a sample of intrinsic noise:

$$P_{2}=1-(1-2\delta )F[\frac{\sigma_{thres}^{2}}{{\left(\sigma_{p}+\Delta \sigma \right)}^{2}}]+\delta ,$$ (8)

The final condition assumes that both sample variances exceed the threshold and the response accuracy is determined by comparing the sample variance of the Pedestal and Staircase image.

$$P_{3}=1-(1-2\delta )F[\frac{\sigma_{p}^{2}}{{\left(\sigma_{p}+\Delta \sigma \right)}^{2}}]+\delta ,$$ (9)

Thus, the NIO model with a hard sensory threshold (henceforth referred to as NIOt) is represented by Eqs 7–9. Note that the same number of free parameters are needed for both versions (NIO and NIOt) of the ideal observer model.

We estimated the parameters of the models by maximizing their likelihoods of having produced the observed responses for each image class. Next, we simulated the models using the best fitting parameter estimates and using a wide range of Δ*σ* and *σ*_p_. The predicted JND for each level of *σ*_p_ is then equal to Δ*σ* at a constant 75% correct. Note that in the original formulation of the models, the parameter *M* represents a discrete number of elements sampled from the stimulus. Participants sampling efficiency can then be determined by dividing *M* by the total amount of elements present in the stimulus. However, since our stimuli consist of continuous texture patterns without well-separated individual Gabor-elements, observers cannot rely on a discrete set of elements for the estimation task. Instead, *M* indicates, on an arbitrary scale but comparable across stimulus classes, the magnitude of the sample used for variance estimation.

## Results

### Experiment 1: Coding of position information with naturalistic stimuli

**Fig 5:**
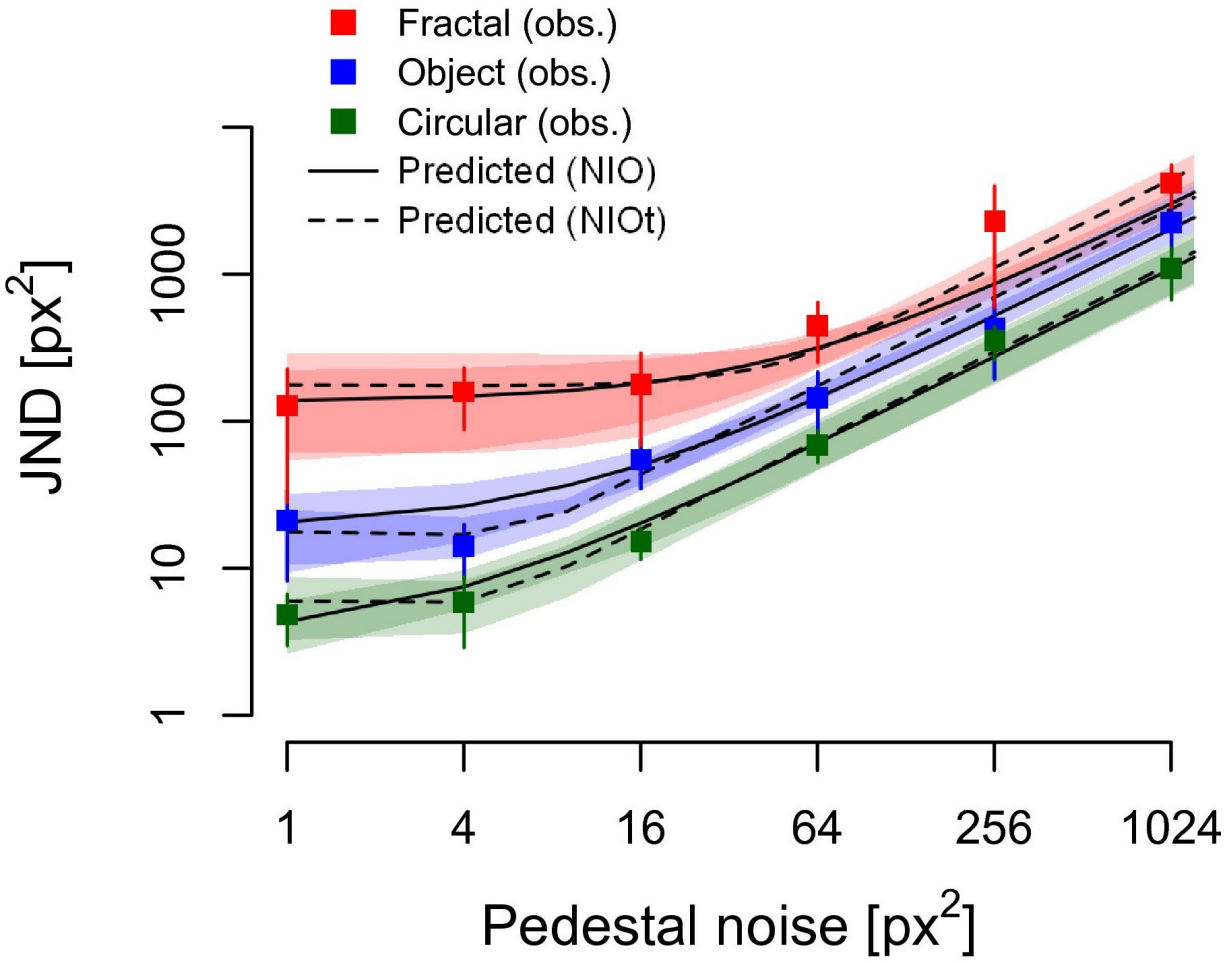
Mean observed and predicted JNDs for position noise discrimination (*N* = 8). Predictions (averaged across all individual fits) from the noisy, inefficient observer model (see Methods) are represented by the lines. Error bars and shaded regions indicate 95% confidence intervals around the mean of the observed and predicted JND, respectively.

We assessed how precisely participants (*N* = 8) encode position information of stimuli that belong to classes of objects, fractals or circular patterns (see Fig 1). The grand-average of the observed JNDs are shown in Fig 5.

We found that the observed values of JNDs at the same pedestal level were significantly different across the image classes (repeated-measures 2-way ANOVA, *F*(2,18) = 33.43 *p <* .001, *η*^2^ = .788). Additionally, planned post hoc pairwise comparisons revealed that the JNDs differed systematically from each other. They were highest for fractals, intermediate for objects, and lowest for circular patterns (object versus fractals: 95% CI for difference [8.76, 15.69], *p* < .001; objects versus circular patterns: 95% CI for difference [1.00, 8.24], *p* = .018; fractals versus circular patterns: 95% CI for difference [10.18, 23.51], *p* < .001).

We quantified the JNDs for each image class and participant, with the noisy, inefficient (but otherwise ideal) observer model (see Methods). As with blur and contrast discrimination [37–40], previous studies of orientation noise discrimination found that the JND for orientation noise was best accounted for by allowing a sensory threshold in the model, below which externally added variance was discounted [23,24,41]. To quantitatively investigate if this is the same for position noise, we employ the model both with and without the added sensory threshold, termed NIOt and NIO, respectively. We found that the predicted JNDs of both models were close to the observed ones (see Figures C and D in S1 File). When testing each of the 24 individual conditions (8 participants, 3 image classes) with Persons’s product-moment correlations between predicted and observed JNDs (*α* = 0.05, *df* = 4), 22 were significant for the NIO and 21 were significant for the NIOt. Moreover, both models fitted the response patterns almost identically (the maximum likelihood after fitting the models favoured the NIO model in 4 out of the 8 participants). Note that the predicted JNDs are not based on fitting the observed JNDs (squares in Fig 1) directly, but on maximum likelihood fits to each participant’s response (correct: “1” or wrong: “0”) on each trial (see Methods).

The systematic differences in observed JNDs between image classes were accounted for by the parameters of both models (see Table 1). There was a main effect of image class on sampling size *M* (repeated-measures 1-way ANOVA; NIO: Sphericity assumed, *F*(2,14) = 16.28, *p* < 0.001, *η*^2^ = .70; NIOt: Sphericity assumed, *F*(2,14) = 19.19, *p* < 0.001, *η*^2^ = .73). In addition, planned pairwise comparisons showed that the average sampling size for circular stimuli was significantly higher than for both objects (NIO: 95% CI for difference [0.87, 3.80], *p* = 0.007; NIOt: 95% CI for difference [1.05, 3.64], *p* = 0.004) and fractal stimuli (NIO: 95% CI for the difference [1.57, 4.54], *p* = 0.002; NIOt: 95% CI for difference [1.39, 4.13], *p* = 0.002). Although sampling sizes for objects were higher than for fractal patterns for all participants, the mean difference was not statistically significant (NIO: 95% CI for difference [-0.20, 1.33], *p* = 0.124; NIOt: 95% CI for difference [-0.15, 0.98], *p* = 0.127) due to one participant having much higher sampling size for objects than the remaining participants (> 2.4 SD). After excluding this outlier from the ANOVA, the mean sampling size for objects was significantly higher than for fractals (NIO: 95% CI for difference [0.08, 0.42], *p* = 0.012; NIOt: 95% CI for difference [0.01, 0.36], *p* = 0.046).

**Table 1:** Best fitting estimated parameter means and SD (in parentheses) of the NIO model with and without a sensory threshold. *M* = size of the sample; *σ*_int_ = internal noise; *σ*_thres_ = sensory threshold noise.

	No threshold (NIO)		With threshold (NIOt)	
	*M*	*σ*_int_	*M*	*σ*_thres_
**Position coding**				
Objects**	3.93 (0.72)	2.27 (1.72)	3.70 (0.60)	2.70 (1.19)
Objects*	3.95 (0.94)	2.85 (1.39)	3.62 (0.68)	2.17 (0.60)
Fractals*	3.39 (0.13)	6.40 (3.66)	3.20 (0.13)	5.53 (2.93)
Circular*	6.28 (1.78)	1.77 (0.73)	5.96 (1.62)	1.78 (0.51)
**Orientation coding**				
Objects**	29.45 (28.73)	18.89 (5.69)	8.41 (3.69)	9.70 (1.79)
Objects***	53.00 (17.34)	17.86 (2.25)	-	-
Fractals***	9.00 (2.64)	15.03 (1.87)	-	-
Circular***	25.00 (5.12)	10.78 (1.12)	-	-

*Based on fitting the JNDs from Experiment 1 (Fig 5)

**Fig 6:**
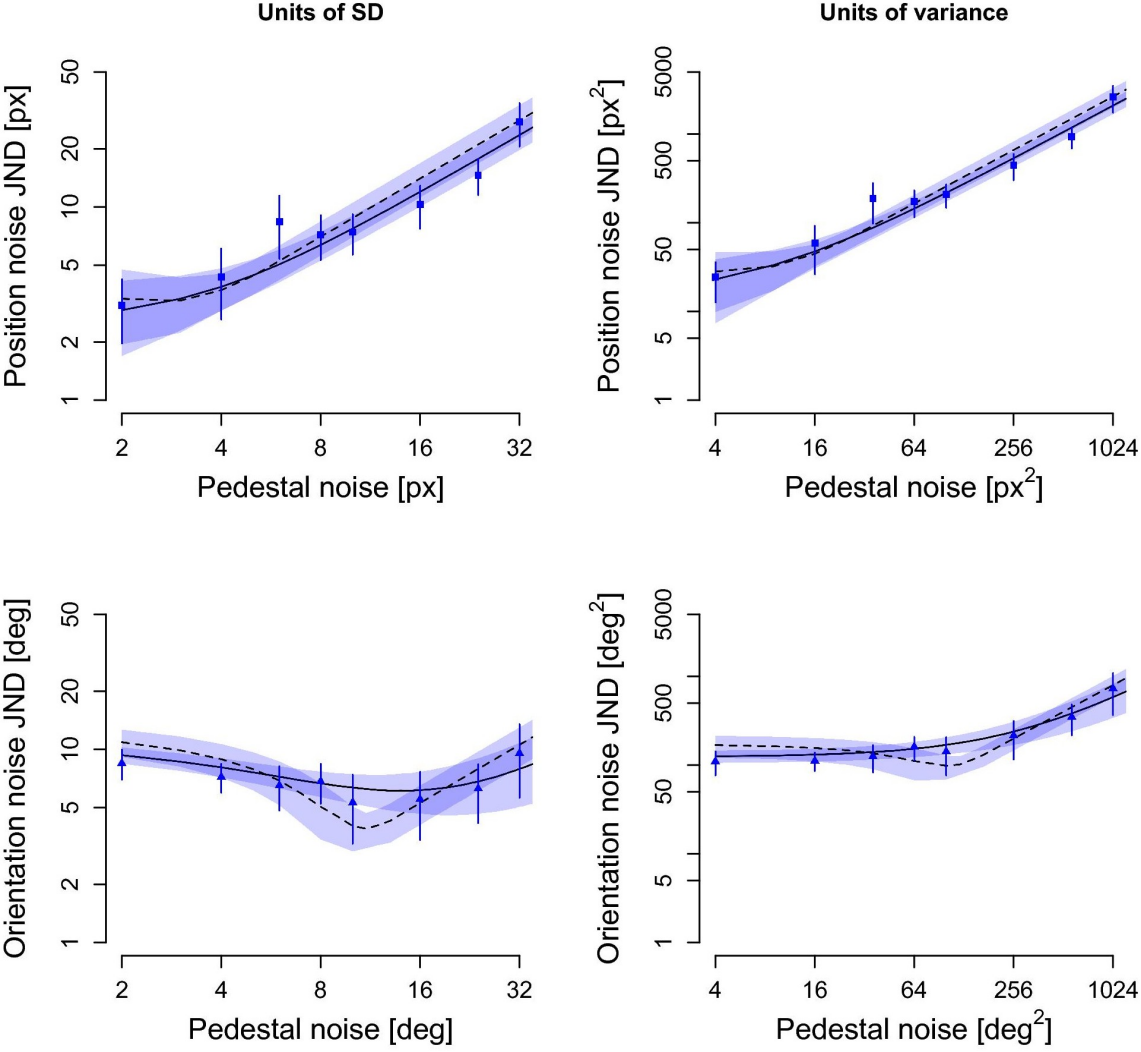
Mean observed and predicted JNDs for position and orientation noise in Experiment 2. The JNDs are presented in SDs (left column) and in variances (right column). Symbols (circles and triangles) represent averaged JNDs across all 8 participants, and the error bars indicating the 95% CI of the mean. Predictions of the ideal observer is represented by the solid (NIO model) and dashed (NIOt model) lines, and the shaded region around the predictions indicate 95% CI of the mean prediction across participants.

**Based on fitting the JNDs from Experiment 2 (Fig 6)

***Re-printed from Table 2 of Christensen, Bex, & Fiser [23]

The internal noise and sensory threshold (see Table 1) were also significantly modulated (in reverse order) by image class (repeated-measures 1-way ANOVA; NIO: Sphericity assumed, *F*(2,14) = 7.61, *p* = 0.006, *η*^2^ = .52; NIOt: Greenhouse-Geisser corrected, *F*(2,14) = 9.68, *p* = 0.015, *η*^2^ = .58). Planned pairwise comparisons revealed that mean internal noise and sensory threshold were highest for fractals, intermediate for objects, and lowest for circular patterns. This was statistically significant between fractals and objects (NIO: 95% CI for difference [0.08, 7.02], *p* = 0.046; NIOt: 95% CI for difference [0.77, 5.95], *p* = 0.018), and fractals and circular patterns (NIO: 95% CI for difference [0.99, 6.51], *p* = 0.015; NIOt: 95% CI for difference [0.64, 5.38], *p* = 0.020), but not between objects and circular patterns (NIO: 95% CI for difference [-0.21, 0.99], *p* = 0.170; NIOt: 95% CI for difference [-0.33, 1.59], *p* = 0.163).

In summary, we found that the precision of position coding of naturalistic stimuli was well accounted for by an ideal observer with internal additive noise and sparse sampling corroborating previous research that used simple and artificial stimuli composed of isolated elements [36]. We also found that the model parameters indicated a large systematic change in the JNDs that was dependent on the complexity (circular patterns versus objects and fractals) and familiarity (objects versus fractals) of the stimulus similarly to earlier results with orientation coding [23]. Finally, extending and re-parameterizing the NIO model to allow for a sensory threshold did not improve the overall likelihoods of the predictions for position noise.

### Experiment 2: Comparing coding of position and orientation information

The result of the first experiment suggests that position information might be encoded without a sensory threshold, which is at odds with earlier findings that used identical methodology and stimulus material, but perturbed each Gabor-element of the images with *orientation* noise instead of position jitter [23]. This discrepancy raises the possibility that orientation and position perturbations are encoded differently in the human visual system. To test this hypothesis, we measured sensitivity to position and orientation noise of the same participant (*N* = 8) at the same time by using a within-group design but otherwise identical task procedure as in Experiment 1 (see Methods). Fig 6 displays the observed JNDs using both standard deviation and variance as the units, and with the predictions of the NIO and NIOt model (see Methods).

For the position noise condition, the NIO model was preferred in 4 participants, but in the orientation noise condition the NIO model was only preferred in 2 out of the total 8 participants based on the likelihood values of the fits.

Transforming units of SD to variance changes the shape of the JND curve (see Methods), reducing the prominence of a dip in the JNDs for orientation noise (left versus right panels in Fig 6). Despite this transformation, the characteristics of the JNDs for the two attributes (upper versus lower panels of Fig 6) remained quite distinct. First, the “masking” region, i.e. the range of pedestal levels resulting in a monotonic increase of the JND with a slope of approximately 1 (Weber’s law), begins around a pedestal variance of 4–16 for position noise and around 64–256 for orientation noise. Second, the JNDs at small pedestals are 2–4 times higher for orientation noise than for position noise. These characteristics indicate a dissociation between the JND for position versus orientation noise as pedestal noise increases, which is directly reflected by the best fitting parameter estimates of the predicted JNDs presented in Table 1. The internal noise and the sensory threshold were both much higher for orientation coding than for position coding for every participant (two-tailed paired t-tests, NIO: 95% CI for difference [10.94, 22.31], *t*(7) = 6.91, *p* < 0.001, Cohen’s *D* = 3.95; NIOt: 95% CI for difference [5.30, 8.70], *t*(7) = 9.77, *p* < 0.001, Cohen’s *D* = 4.60). Mean sampling size was likewise significantly higher for orientation noise than position noise (two-tailed paired t-tests, NIO: 95% CI for difference [1.85, 49.19], *t*(7) = 2.55, *p* = 0.038, Cohen’s *D* = 1.26; NIOt: 95% CI for difference [1.91, 7.52], *t*(7) = 3.98, *p* = 0.005, Cohen’s *D* = 1.78).

In sum, we confirmed that the JNDs for position noise are different than JNDs for orientation noise not only by a scaling factor but also by how they evolve across increasing levels of pedestal noise. In addition, the fact that 6 out of 8 participants had better fits with the NIOt model when tested with orientation noise suggest that local-elements’ orientation is encoded with a sensory threshold.

### Experiment 3: Coding of combined information

The parameters in Table 1 suggest that differences in coding between position and orientation information are not merely a matter of differences in overall sensitivity to the two attributes. If the latter were the case, we would expect coherent changes in the parameters of both the NIO and NIOt models: if sampling efficiency (*M*) *increases*, internal noise (*σ*_int_) and sensory threshold (*σ*_thresh_) should *decrease* and, thus, enhance sensitivity. Instead, all three parameters are higher for orientation coding than for position coding (Table 1), which suggest different underlying encoding mechanisms for the two attributes. If so, the visual system might handle the two kinds of information independently. We explored this possibility in Experiment 3 by measuring JNDs in a mixed noise condition (see Methods). Participants (*N* = 9) discriminated between stimuli perturbed by either position-noise-only or orientation-noise-only (single-noise conditions), or by an equal combination of both position and orientation noise. If the same mechanism encodes both position and orientation information, the JNDs in the combined condition should either match the JND for the attribute with the higher value (i.e. the coding precision of one attribute determines the JND for combined noise) or it should degrade further (i.e. adding noise to a second attribute should hinder discrimination performance more and elevate JNDs for the combined noise). On the other hand, if each attribute is treated independently, then the JNDs for the combined noise should improve since each attribute could serve as independent cues for the variance discrimination task. Thus, exploiting the double dissociation in the direction of changes in the combined noise condition can shed light on whether orientation and position are encoded as separate attributes.

**Fig 7:**
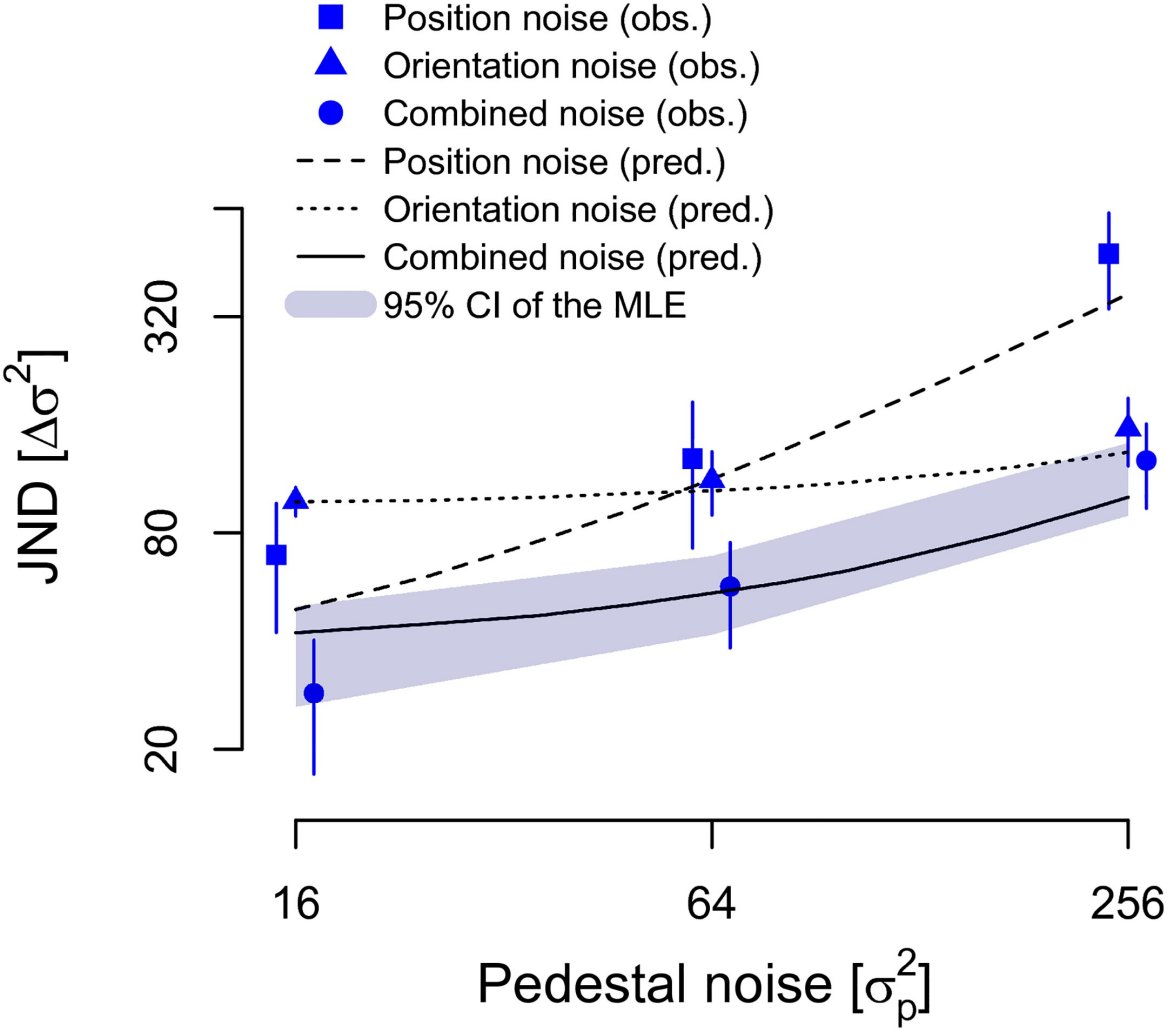
Mean observed and predicted JNDs for position noise (squares), orientation noise (triangles), and an equal combination of position and orientation noise (circles). Predicted noise for position (dashed line), orientation (dotted line) and combined noise (solid line) were derived from the NIO model without threshold, error bars denote the 95% CI. The 95% confidence interval of a maximum likelihood estimate of the JNDs for the combined noise (grey shaded area) was calculated on the basis of the individually measured JNDs for position and orientation noise. Both the observed (circles) and predicted (solid line) JNDs for combined noise fall well within the 95% confidence interval of a maximum likelihood observer (grey shaded region) based on JNDs from the position-noise-only and orientation-noise-only conditions. Individual data points were slightly jittered around the pedestal levels for display purposes.

The observed JNDs (Fig 7) for the single-noise conditions were well predicted by the NIO model, and the results replicated the findings of the previous experiments: Coding of local element’s orientation was subjected to much higher internal noise and sampling efficiency than coding position (see Table 2).

**Table 2:** Best fitting estimated parameter means and SD (in parentheses) of the NIO model without a sensory threshold to stimuli from the object image class.

Noise type	*M*	*σ*_int_
Position noise	5.01 (2.66)	4.34 (3.73)
Orientation noise	113.74 (67.43)	32.07 (12.09)
Combined noise	49.59 (42.89)	12.45 (6.79)

The difference in parameter estimates quantified a significant interaction effect of the observed JNDs for position and orientation noise (Greenhouse-Geisser corrected, 2-by-3 repeated measures ANOVA with noise type (position, orientation) and pedestal levels as factors yielding *F*(1.26,10.09) = 25.76, *p* < 0.001, *η*^2^ = 0.76).

Crucially, when local elements were perturbed with orientation and position noise simultaneously, discrimination sensitivity improved significantly over both orientation-noise-only and position-noise-only conditions (Fig 7). A 3-by-3 repeated measures ANOVA with noise type (position, orientation, combined) and pedestal levels as within-subject factors showed a main effect of noise type (Greenhouse-Geisser corrected *F*(1.07,8.53) = 27.68, *p* = 0.001, *η*^2^ = 0.78), and planned pairwise comparisons showed that the JNDs for combined noise were lower than for position noise (95% CI for difference [92.81, 217.01], *p* < 0.001) and orientation noise (95% CI for difference [37.40, 65.21], *p* < 0.001). Moreover, the level of JND enhancement for each pedestal level in the combined condition was not discriminable from what optimal combination (i.e. weighted average) would predict based on the coding precisions of the individual attributes (repeated measures ANOVA between observed JNDs for combined noise and JNDs predicted by MLE, with pedestal levels as factors. Greenhouse-Geisser corrected: *F*(1,8) = 0.043, *p* = 0.841). Further, the Bayes factor for related samples t-tests supported this null effect at low pedestal levels (1.36), and strongly supported the null effect at pedestal level 64 (4.08) and 256 (3.51). Specifically, the observed and predicted JNDs for combined noise both fell well within the 95% CI for the MLE of Eq 5 for in tested pedestal levels.

### Experiment 4: Automatic encoding of variances for different attributes

**Fig 8:**
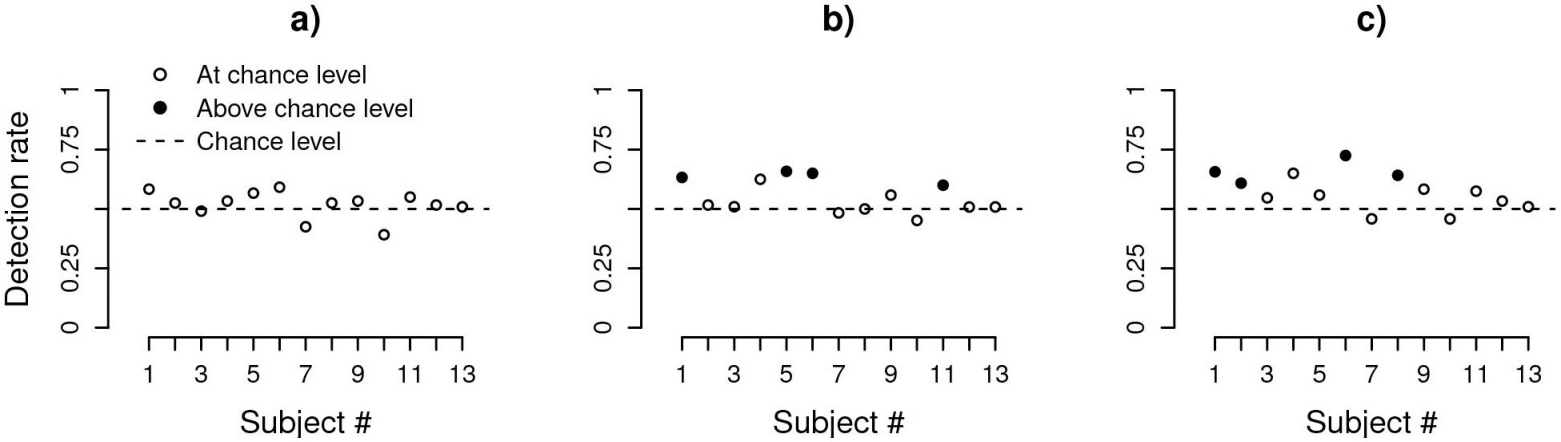
Detection rates for all participants (N = 13) for correctly detecting an odd-one-out stimulus. The odd-one-out stimulus was presented in a stream of 5 consecutive presented stimuli with *σ*, the noise level in SDs, equal to (A) 4, (B) 8, and (C) 16. Circles represent individual participants performing at chance level (open circles) or above (solid circles) according to the exact binominal test.

The comparison of the JND for position and orientation noise faces one potential problem: there is no direct mapping between the perceptual effects of the two noise types despite similar physical metrics. For example, it could be that orientation noise and position jitter create radically different visual stimuli leading to different amount of perceived perturbations, which would confound our claim that coding of the two attributes are distinct, independent, and automatic. Even though simple visual inspection of the stimuli does not support this alternative (see Methods), we conducted Experiment 4 (*N* = 13) to investigate how well a stimulus perturbed with one noise type was detected among stimuli perturbed with the other noise type for 3 different noise levels (see Methods). Fig 8 shows the detection rates for each participant (321–395 trials, SD = 17 trials) separated by noise level.

Overall, the detection rates were around chance level (mean 54.9% SD 6.3%) indicating that participants were unable to distinguish reliably between the two noise types despite their conscious effort and the inevitable within-experiment learning effect induced by 53–65 repetitions per experimental condition. We formally tested this conclusion by evaluating each participant’s responses separately using the exact binomial one-tailed test with a null hypothesis of 50%. When evaluating across both noise types, we found that 0/13 for low noise; 4/13 for intermediate noise; and 4/13 participants for high noise tested significantly (see solid points in Fig 8). This result indicates that, at each level of noise used in the main experiments, the majority of participants cannot explicitly assess what attribute was perturbed. Therefore, the differences found in Experiment 2 and the independent integration of variance information demonstrated in Experiment 3 cannot be explained by large-size perceptual differences in the magnitude of perturbations at the same pedestal level of the orientation and position attributes.

## Discussion

We combined a novel stimulus generation technique with a simple 2AFC task to assess patterns of sensitivity to variability of low-level attributes in naturalistic images. With this method, we separated the effects of higher-order configural content and prior expectancy without introducing confounding differences in low-level stimuli characteristics (see Figures A and B in S1 File). Importantly, our results are highly compatible with and build on previous reports using simple and artificial stimuli [18,24]. Our present results support three conclusions that shape our view on the general process of visual perception.

First, coding of local low-level position and orientation information, which are essential attributes for e.g. contour integration [11,14,42,43], depend strongly on high-level processes as demonstrated by image-class-dependent differences in coding sensitivity (Fig 5; Table 1). This suggests that structure-based expectations and experience-based interpretations operate in tandem with low-level spatial sensory information during attribute coding. Since all image classes consisted of the same number and same global distribution of local elements, these image-class-dependent differences cannot be explained by low-level differences among the stimuli. Instead, we propose that these effects emerge because objects and fractals in this task are processed with relatively less reliance on the sensory input. Indeed, circular patterns yield JNDs for position noise very similar to those in earlier reports [9,18,36]. This is because circular patterns are conceptually the closest to isolated Gabor stimuli, as they possess a relatively simple and nonspecific local structure. At the same time, these stimuli do not invoke strong top-down local expectancy of feature configuration due to lack of specific higher-order visual features and familiarity. Thus, the computation of both position noise and orientation noise [23] estimates in the circular patterns are dominated by the precision of sensory input.

In stimuli from the object and fractal image classes, the effect of between-elements top-down constraint is stronger due to a comparatively more complex structure with long and disjointed edges, corners, etc. In addition, top-down expectancy due to familiar higher-order statistics associated with objects also factors in to the processing of the stimuli from the object class (e.g. a camera with a lens is supposed to have a particular configuration) but is lacking for the unfamiliar fractals. These top-down effects are then combined with less detailed coding of the sensory input compared to circular patterns, where top-down activation is assumed to be less dominant [44]. Such interpretation of the role of familiarity with higher-order image statistics is in agreement with previous studies showing that practice, i.e. improving familiarity, can change position jitter sensitivity by re-tuning an observer’s template [18]. The notion that processing of object and fractal images relies less on sensory input is supported by evidence showing reduced activity in primary visual cortex for object-like stimuli, when compared to simple random lines [22], and increased top-down activity for both object and fractals [44].

Our second conclusion is that despite apparent similarities in the stimuli (Fig 1), and even though they seem to have no explicit awareness of stimulus differences (Experiment 4), humans encode position and orientation information in markedly distinct manner (Figs 6 and 7). When fitted with an ideal observer model, we found that internal noises and sampling efficiencies were significantly lower for position information than for orientation information for visually almost indistinguishable images. Since the internal noise effectively discounts externally added noise below its value, this suggests that coding of orientation information is subjected to a stronger threshold-like mechanism than coding of position information. This is also why the JNDs for orientation noise in Experiment 2 were better predicted in 6 out of 8 participants when a sensory threshold was assumed (see Fig 6).

Our third conclusion is that not only positions and orientations of small image segments are encoded differently, but that these two types of information are handled independently. In fact, our measure with stimuli corrupted by combined noise uncovered that humans integrate information about the two types of noise in a close to optimal manner, as sensitivity to combined noise was optimally improved based on the reliability with which each attribute was coded in isolation (Fig 7). Until now, probabilistic combination of perceptual information was demonstrated either between modalities [33] or within modalities [45], but always for cognitively identifiable distinct sources of information. Our finding indicates that the underlying computational strategy might be much more pervasive in the visual system reaching to the lowest level of visual attributes represented in the cortex.

To accommodate these results, we propose a new take on orientation and position processing based on two notable architectural differences between the neural representation of those two attributes. First, while cortical tissue under an area of cortical surface in the primary visual cortex (i.e. a cortical hyper-column) can provide evidence for only one position, namely whether the feature is in that position or not, the same cortical column can support hypotheses of different orientations or even multiple orientations [21,46]. Thus, while different position information can be combined only across cortical columns, combining different orientation information has an extra level of complexity of dealing with potentially contradicting evidence arriving from within each column in addition to across columns. Second, there is a difference in sensitivity to position and orientation in complex cells within the striate cortex [47]. While both simple and complex cells are comparably tuned for orientation, complex cell responses are invariant of the position of an oriented line within the receptive field, whereas orientation is represented more specifically [47]. While the exact functionality and implementation of processing orientation or position information are presently unknown, based on the above differences, it is reasonable to assume a different and more complex computational mechanism for orientation processing.

**Fig 9:**
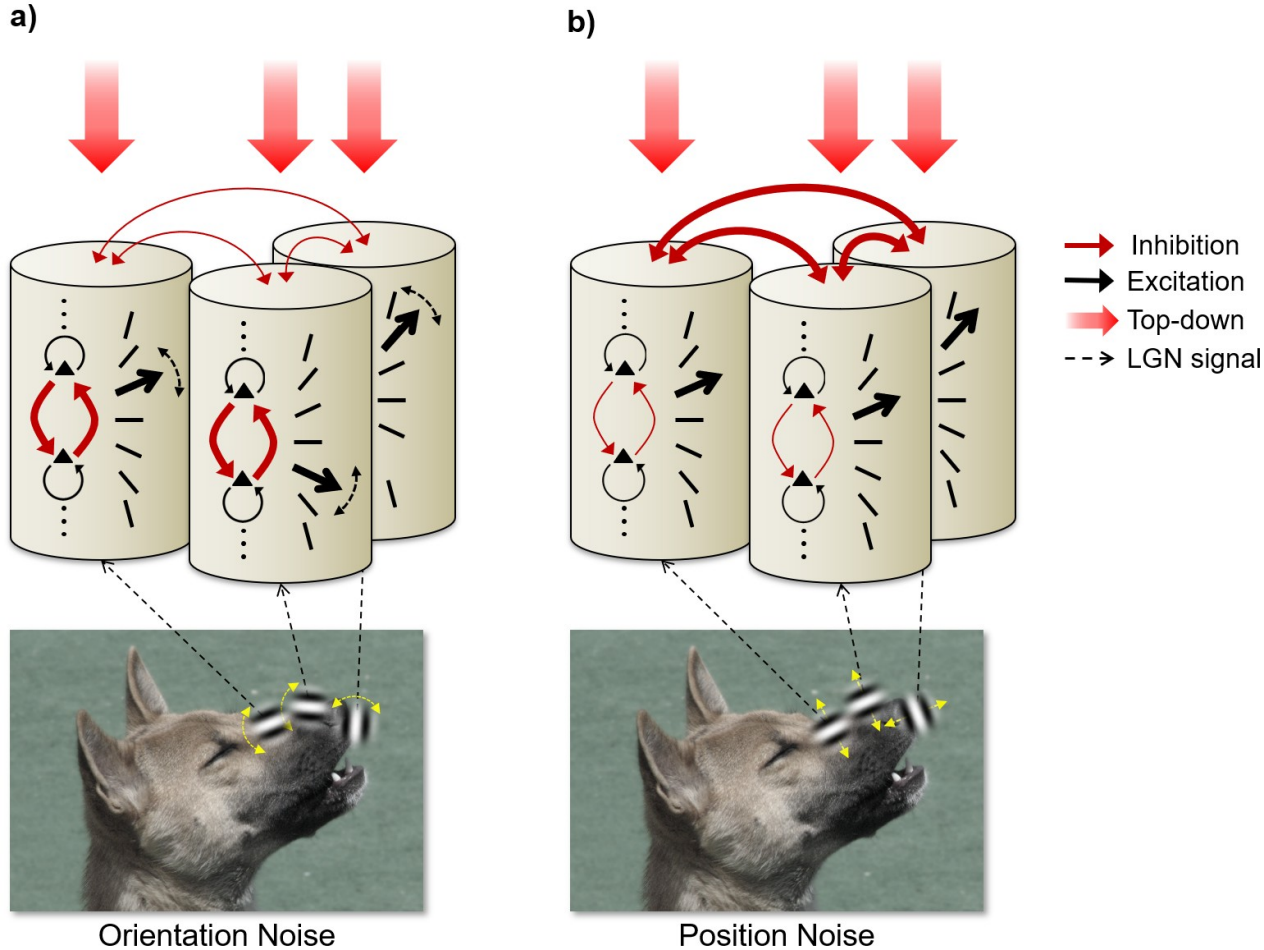
Cartoon illustrating coding of three low-level features in a naturalistic image. Low-level features, represented by contour segments, are perturbed on either (A) their orientation attribute or (B) their position attribute. In each panel, at the bottom, the visual stimulus is seen with the three perturbed elements (Gabor patches), and the nature of perturbation (yellow double arrows). Above, three hyper-columns of V1 are depicted (cylinders) with orientation selective cells (black triangles), and with the LGN input from the corresponding visual segments the columns process (black dashed arrows). The strength of the within-column inhibitory (red arrows) and excitatory (black arrows) recurrent connections between cells is represented by the thickness of the arrows. In each column, one of the possible orientations of the underlying image segment (column of oriented bars) is indicated as the most feasible interpretation of the present input (thick arrow). Between-column interactions are represented with red double arrows among the three columns, while top-down effects are depicted by the red downward arrows. (A) For coding of features with orientation noise, the selected orientation of each hypercolumn (thick arrow) is computed by recurrent interactions between orientation-tuned cells within the column. Top-down effects influence the coding resolution (dashed double arrow) by modulating the strength of inhibition between competing orientations within each column. In turn, weaker inter-columnar lateral connections help to compute a global estimate of orientation noise in the visual scene. (B) For coding of features with position noise, position identification requires linking Gabor-elements and columns that match the feature’s expected position. Added position noise does not interfere with coding of orientation within each column (weak recurrent within-column arrows) resulting in easily computed well-articulated within-column orientation information (black arrow). Instead, top-down effects heavily modulate the inter-columnar associations (thick inter-column arrows) and the resulting strong expectations can prevent elements from being coded.

We propose that this more complex mechanism indeed implements a computation that essentially amounts to applying a threshold under particular tasks [23,38]. The underlying idea of a threshold is to deliberately omit coding information below a (dynamically) set magnitude in order to discard information that is highly likely to be irrelevant [23,48,49]. The idea that noise is detected only when it exceeds a threshold is supported by previous studies of blur discrimination [39,40]. Such a threshold might be implemented as intra-columnar recurrent excitation and inhibition among orientation selective units with its strength determining the resulting encoding resolution [50–54], which can then be combined across columns through inter-columnar connections. In this scheme, depicted in Fig 9, intra-columnar processes (i.e. internal noise) prevent some local information to be coded (Fig 9A).

In line with our proposed view on intra-columnar “thresholder”, Gur et al. [54] suggested that orientation tuning in V1 of alert monkeys is shaped by interactions between excitatory and inhibitory units across layers of V1 and that strong inhibition leads to sharp tuning. Moreover, Ringach et al. [21]emphasized that, in addition to intricate local inhibition and excitation, orientation selectivity in macaque V1 is a dynamically emerging feature strongly shaped by global suppression, attesting to our proposal that top-down effects act directly on the level of low-level orientation coding.

In contrast, position noise discrimination requires integrating information only across hyper-columns based on the retinotopic mapping and on position-invariant responses of complex cells. Here, top-down effects would influence inter-columnar processes and integration across position insensitive complex cell responses to determine the expected position of each Gabor with respect to particular columns and receptive fields. Such a scheme, illustrated in Fig 9B, would produce the Weber’s law-like function with the JND simply rising with the addition of noise [55,56]. While this proposal of ours is admittedly speculative, there exist indirect hints in the literature supporting our psychophysical finding that position and orientation information is indeed independently coded. For example, Bosking et al. [57] demonstrated that, even in tree shrews, while the position and orientation of a thin line presented across the visual field are orderly mapped in V1 at a fine scale, the mapping of these attributes have no local relationships between them.

In conclusion, using novel stimuli and a noise discrimination task, we found top-down modulations and differences in psychophysical measurements of low-level position and orientation coding in naturalistic images. This argues for a more adaptive and yet more specialized dynamic perceptual process compared to the traditional filter-based feed-forward model of human vision.

## Supporting information

S1 File **Gabor orientation distribution (Figure A)**. Density of Gabor orientations across all 40 stimulus exemplars grouped by image class (panels from left to right) and spatial frequency band (panels from top to bottom). **Stimuli and image statistics (Figure B)**. a) average amplitude spectrum and SD across all source images (black) and stimuli (purple) from each image class. The radial average is based on the amplitude of the spatial frequency decomposition. It is computed for each stimulus class (separate panels) and for both the source images (black lines) and the synthesized stimuli (purple lines). Circular patterns were synthetically generated; thus, these stimuli had no source images. b) spatial density profiles of stimuli from each image class separated for spatial frequency and binned into circular bands with increasing radial distance from element centres. The bands were 0.25λ_s_ wide. The shaded area around each density profile represents SD. **Observed and predicted JNDs for position noise discrimination (Experiment 1, participant S1–S4) (Figure C)**. The predictions are based on fitting the model with a sensory threshold (NIOt, dashed line) and without a sensory threshold (NIO, solid line). Error bars indicate the 95% confidence intervals produced by bootstrapping each experimental condition (see Methods in the main manuscript). **Observed and predicted JNDs for position noise discrimination (Experiment 1, participant S5–S8) (Figure D)**. The predictions are based on fitting the model with a sensory threshold (NIOt, dashed line) and without a sensory threshold (NIO, solid line). Error bars indicate the 95% confidence intervals produced by bootstrapping each experimental condition (see Methods in the main manuscript).
(DOCX)
