# Correction: Coding of low-level position and orientation information in human naturalistic vision

**DOI:** 10.1371/journal.pone.0220502

**Published:** 2019-07-25

**Authors:** Jeppe H. Christensen, Peter J. Bex, József Fiser

## Notice of republication

Incorrect versions of Fig 1 and Fig 9 were published in error. This article was republished on July 17, 2019 to correct for this error. Please download this article again to view the correct version.
