# Supplementary material for: Coding of low-level position and orientation information in human naturalistic vision
Source: PLoS One. 2019 Feb 11;14(2):e0212141. doi: 10.1371/journal.pone.0212141 (PMC6370245; doi:10.1371/journal.pone.0212141)
Supplement: S1 File — Gabor orientation distribution (Figure A). Density of Gabor orientations across all 40 stimulus exemplars grouped by image class (panels from left to right) and spatial frequency band (panels from top to bottom). Stimuli and image statistics (Figure B). a) average amplitude spectrum and SD across all source images (black) and stimuli (purple) from each image class. The radial average is based on the amplitude of the spatial frequency decomposition. It is computed for each stimulus class (separate panels) and for both the source images (black lines) and the synthesized stimuli (purple lines). Circular patterns were synthetically generated; thus, these stimuli had no source images. b) spatial density profiles of stimuli from each image class separated for spatial frequency and binned into circular bands with increasing radial distance from element centres. The bands were 0.25λs wide. The shaded area around each density profile represents SD. Observed and predicted JNDs for position noise discrimination (Experiment 1, participant S1–S4) (Figure C). The predictions are based on fitting the model with a sensory threshold (NIOt, dashed line) and without a sensory threshold (NIO, solid line). Error bars indicate the 95% confidence intervals produced by bootstrapping each experimental condition (see Methods in the main manuscript). Observed and predicted JNDs for position noise discrimination (Experiment 1, participant S5–S8) (Figure D). The predictions are based on fitting the model with a sensory threshold (NIOt, dashed line) and without a sensory threshold (NIO, solid line). Error bars indicate the 95% confidence intervals produced by bootstrapping each experimental condition (see Methods in the main manuscript). (DOCX) [file pone.0212141.s001.docx]

Coding of low-level position and orientation information in human naturalistic vision

Jeppe H. Christensen, Peter J. Bex and József Fiser

**Supplementary information**

# Stimuli image statistics

We compared low-level stimuli attributes and statistics among the image classes by assessing the orientation distribution, the spatial frequency characteristics, and the spatial density profiles.

## Orientation distribution.

The distribution of oriented contours in natural scenes of indoor and outdoor environments have been found to be dominated by vertical and horizontal elements (1), and thus, it has been claimed that human and animal vision is optimized for such characteristic in natural vision. This implies that coding precision, i.e. the JND, should be (on average) better for stimuli that features strong vertical and/or horizontal contours, and differences in this characteristic among image classes could constitute a potential confound. We evaluated the distribution of local elements orientation (individual Gabor-elements) across the three image classes by computing the histograms for each class and spatial frequency across 40 stimulus exemplars. The results are presented in Figure 2.


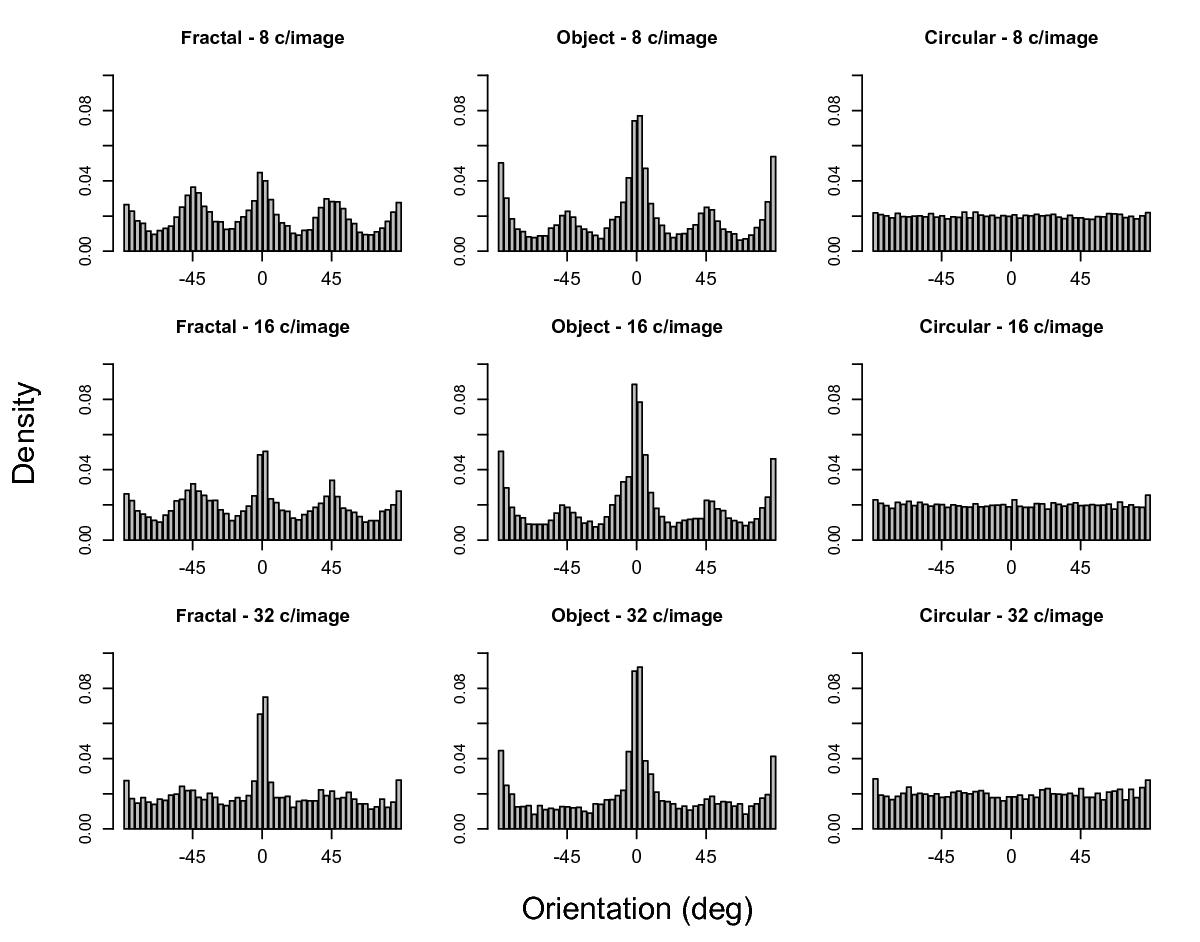


**Figure A:** **Gabor orientation distributions.** Density of Gabor orientations across all 40 stimulus exemplars grouped by image class (panels from left to right) and spatial frequency band (panels from top to bottom).

Both the object and fractal class show characteristics of natural scenes with peaks at horizontal (±90 deg.) and vertical (0 deg.) orientations. This implies that these stimulus classes contain contours and straight edges predominantly at the cardinal orientations. In contrast, the distribution of orientations for circular patterns is uniform, as expected. Nevertheless, our results show (Results) that circular patterns are coded more precise than objects and fractals despite the lack of an excess number of Gabors in the cardinal orientations. Therefore, any difference in the distribution of Gabor orientations among the stimulus classes cannot explain our main findings.

## Frequency analysis

To validate that the power across spatial frequencies were evenly distributed among the image classes we compared the radial average amplitude spectrums (see Figure S2a). We calculated the radial average from the amplitude spectrum of each source image and its associated synthesized stimulus by applying a 2-dimensional discrete Fourier-transform. The resulting radial average amplitude (in squared intensity) are functions of spatial frequencies given in cycles/image with image size equal to 512x512 pixels. To comply with natural stimuli and to not introduce confounding effects, the radial amplitude of each image class should scale approximately with the spatial frequency, *f*, as $const*f^{-\alpha}$ with the slope $\alpha$ close to the range of 1 to 2 (2, 3). Thus, we fitted the spectrum from each stimulus and image with the relationship $amplitude=const*{frequency}^{-\alpha}$ within the range of 6 to 34 cycles/image, which is the range containing most of the power in the synthesized stimuli as a result of our stimulus generation method.

For the source images, the mean slopes (SD) were 1.24 (0.28) and 1.05 (0.27) for objects and fractals, respectively. For the stimuli, the mean slopes (SD) were 1.30 (0.07), 1.34 (0.11), and 1.44 (0.04) for objects, fractals, and circular patterns, respectively. Thus, the amplitude spectrum of the synthesized stimuli shows power scaling comparable to that found in naturalistic images within the relevant range of spatial frequencies.

## Spatial density

Each Gabor-element within each spatial frequency band were placed with a constraint of inter-element spacing of 2σ, were σ = 0.25λ_s_ (λ_s_ = Gabor wavelength at spatial frequency s). This ensured that the image classes on average exhibited similar spatial density profiles. However, despite this method, profiles might have differed between the classes due to the higher-level configuration of the images. For example, in the object class, elements of the stimuli are concentrated around the high-contrast regions of the source images (e.g. edges). We conducted a post-hoc analysis to test for such differences in the spatial density profile between image classes. Specifically, for each Gabor-element of the stimuli from each image class, we counted the number of adjacent elements within a small surrounding circular band (bandwidth 0.25λ_s_). By step-wise increasing the distance of the circular band from the element centre, we computed the function of spatial density, i.e. average number of adjacent elements binned by radial distance. Figure S2b shows the resulting spatial density profiles separated for image class and spatial frequency band. As expected, the spatial density profiles were highly overlapping, and thus, comparable across image classes.


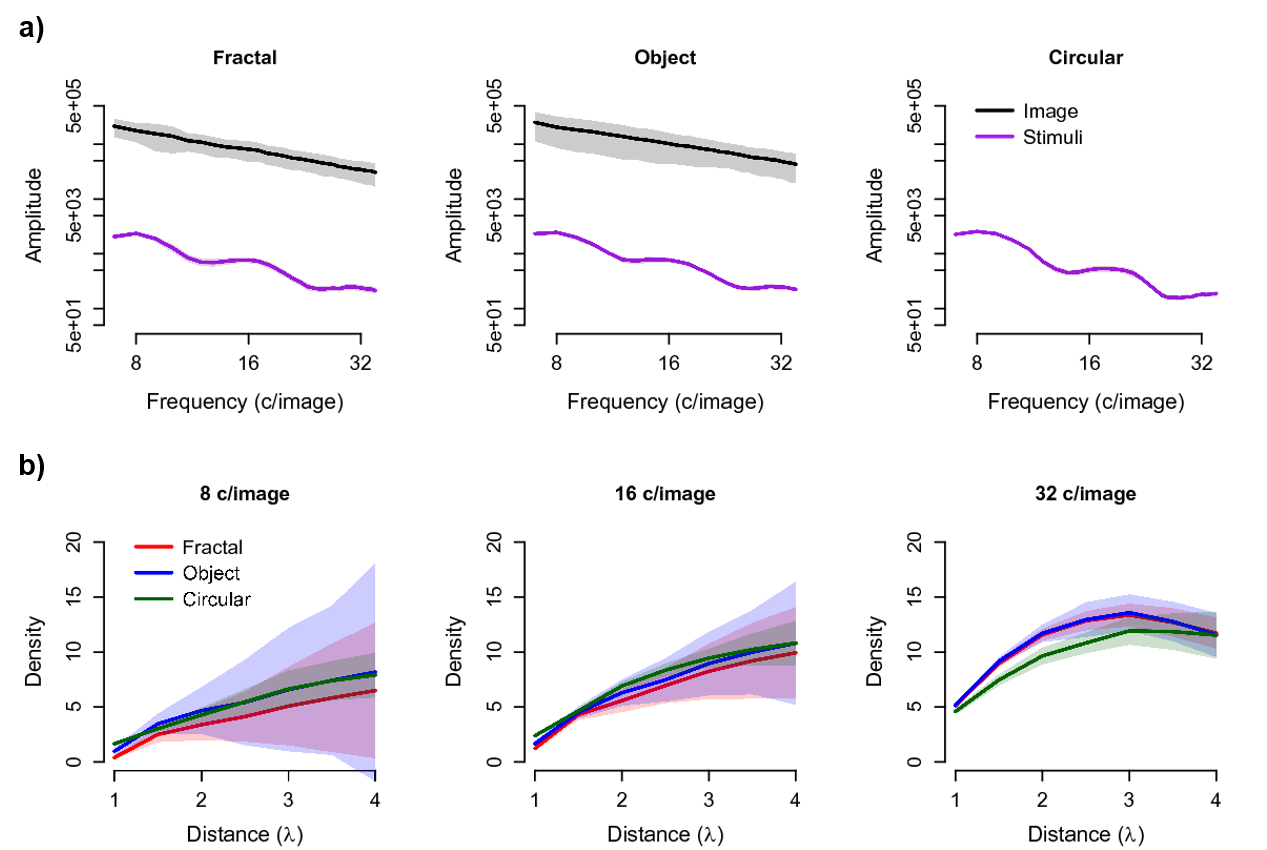


**Fig****ure B: Stimuli and image statistics.** a) average amplitude spectrum and SD across all source images (black) and stimuli (purple) from each image class. The radial average is based on the amplitude of the spatial frequency decomposition. It is computed for each stimulus class (separate panels) and for both the source images (black lines) and the synthesized stimuli (purple lines). Circular patterns were synthetically generated; thus, these stimuli had no source images. b) spatial density profiles of stimuli from each image class separated for spatial frequency and binned into circular bands with increasing radial distance from element centres. The bands were 0.25λ_s_ wide. The shaded area around each density profile represents SD

# Individual fits of the Noisy Inefficient observer model (NIO)


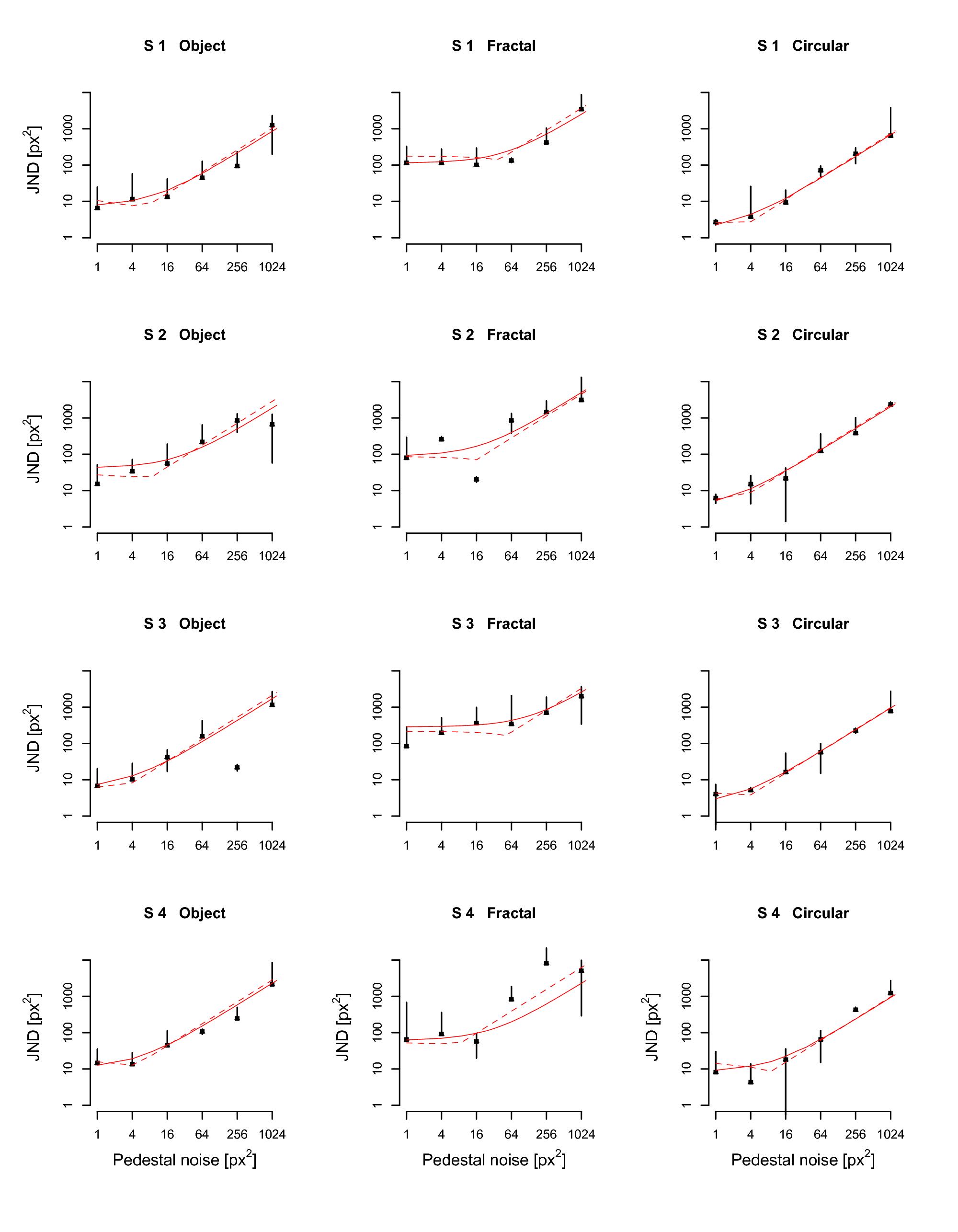


**Figure C: Observed and predicted JNDs for position noise discrimination (Experiment 1, participant S1 – S4).** The predictions are based on fitting the model with a sensory threshold (NIOt, dashed line) and without a sensory threshold (NIO, solid line). Error bars indicate the 95% confidence intervals produced by bootstrapping each experimental condition (see Methods in the main manuscript)

**
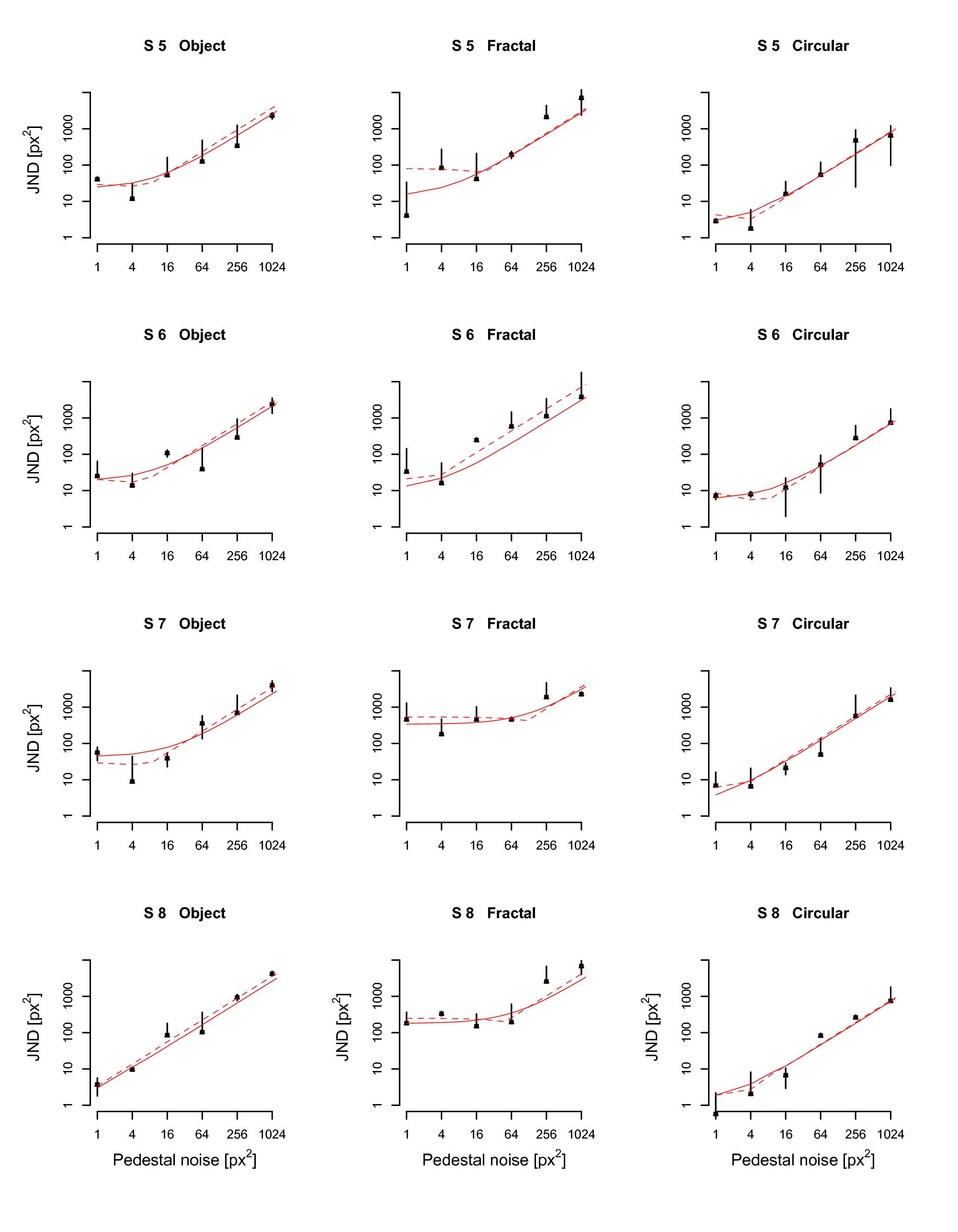
**

**Figure D: Observed and predicted JNDs for position noise discrimination (Experiment 1, participant S5 – S8).** The predictions are based on fitting the model with a sensory threshold (NIOt, dashed line) and without a sensory threshold (NIO, solid line). Error bars indicate the 95% confidence intervals produced by bootstrapping each experimental condition (see Methods in the main manuscript)

**References**

1. Coppola DM, Purves HR, Mccoy AN, Purves D (1998) The distribution of oriented contours in the real world. *Neurobiology* 95:4002–4006.

2. Millane RP, Alzaidi S, Hsiao WH (2003) Scaling and power spectra of natural images. *Proc Image Vis Comput*:148–153.

3. Tolhurst DJ, Tadmor Y, Chao T (1992) Amplitude spectra of natural images. *Ophthalmic Physiol Opt* 12(2):229–32.
